# Chondrosarcoma arising from long-standing Dysplasia Epiphysealis Hemimelica of the proximal humerus: A case report

**DOI:** 10.1051/sicotj/2025057

**Published:** 2026-02-03

**Authors:** Nikolaos A. Stavropoulos, Dimitra P. Papagelopoulos, Olympia Papakonstantinou, Penelope Korkolopoulou, Eleftheria Lakiotaki, Panayiotis J. Papagelopoulos

**Affiliations:** 1 First Department of Orthopaedic Surgery, School of Medicine, National and Kapodistrian University of Athens, Attikon University General Hospital 1 Rimini Street 12462 Chaidari Athens Greece; 2 Second Department of Radiology, School of Medicine, National and Kapodistrian University of Athens, Attikon University General Hospital 1 Rimini Street 12462 Chaidari Athens Greece; 3 First Department of Pathology, School of Medicine, National and Kapodistrian University of Athens 75 Mikras Asias Street 115 27 Goudi Athens Greece

**Keywords:** Dysplasia Epiphysealis Hemimelica (DEH), Trevor's disease, Chondrosarcoma, Cartilaginous, Proximal Humerus

## Abstract

Dysplasia Epiphysealis Hemimelica (DEH), or Trevor’s disease, is a rare, nonhereditary skeletal disorder involving abnormal cartilaginous overgrowth of the epiphysis. To our knowledge, malignant transformation has not been previously documented. We report a unique case of chondrosarcoma arising from a DEH lesion in the proximal humerus nearly 30 years after the initial diagnosis. The patient was treated with wide resection and reconstruction using a proximal humeral replacement with a reverse-constrained total shoulder arthroplasty. This case highlights the need for long-term follow-up in patients with DEH, especially when new symptoms suggest possible malignant transformation.

## Introduction

Dysplasia Epiphyseal Hemimelica (DEH) is an uncommon disorder of cartilage growth characterized by overgrowth mainly in the metadiaphyseal region, resulting in asymmetric development [1–3].

Literature review has revealed a limited number of young adults with such dysplasia [2]. Cases ranging from dysplasia limited to a single epiphysis, in more than one epiphysis but in the ipsilateral limb involved, and diffuse in the limb have been described [2]. Recurrence or transformation to malignancy in adults has never been documented to the best of our knowledge [2]. We present a case of late onset of chondrosarcoma secondary to Trevor’s disease of the proximal humerus.

The patient was informed that the data concerning the case would be submitted for publication, and he provided consent.

## Statement of informed consent

The patient was informed that data concerning the case would be submitted for publication and agreed.

## Case presentation

**Figure 1:**
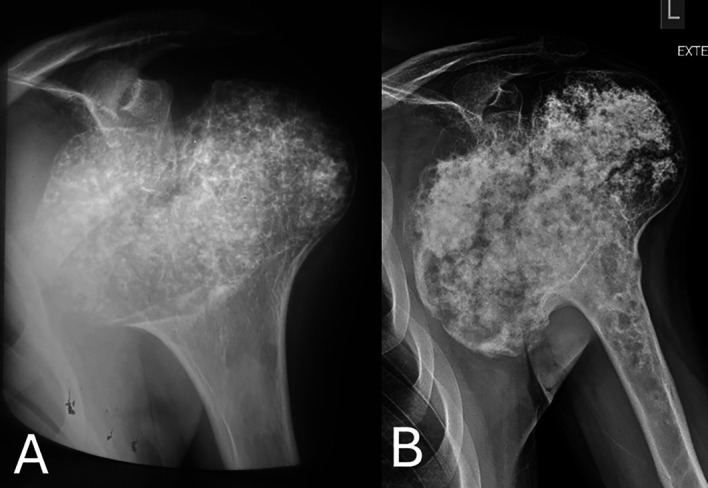
(A) Anteroposterior plain radiograph of left shoulder at the time of initial diagnosis (age 17), showing a bulky bilobed symmetric osteocartilagenous overgrowth of the proximal humeral epiphysis with stippled calcifications, cortical thinning, and a sharp zone of transition towards the metaphysis. (B) Anteroposterior plain radiograph of the left shoulder revealing the bulky, bilobed, incongruent osteocartilagenous overgrowth of the proximal humeral epiphysis. The overgrowth was asymmetric with exuberant enlargement of the medial component, which presented osteolytic areas and areas of cortical breach. Rings and arcs, calcifications, and osteolytic foci were evident in the proximal metaphysis.

A 47-year-old male presented to our Department’s Outpatient Clinic with a painful left shoulder mass. His medical history includes a biopsy-proven DEH (Trevor’s Disease) of his left proximal humerus, regularly followed up over the last thirty years. As per his medical history, the patient sought medical advice following shoulder pain associated with sports activities. Plain radiographs of the shoulder at the time of the initial diagnosis revealed a bulky, bilobed, symmetric osteocartilagenous overgrowth of the proximal humeral epiphysis with stippled calcifications, cortical thinning, and a sharp zone of transition towards the metaphysis. The metaphysis showed remodeling and flaring proximally, but it was unaffected by the osteocartilagenous process (Figure 1A). The patient underwent an open biopsy, which reported a lesion indicative of the developmental disorder of an isolated type. The patient described that the shoulder has enlarged throughout the years, growing bulkier than what is expected by the skeletal maturity, especially recently over a short period.

The patient presented with a recent onset of shoulder pain and restricted shoulder range of motion. He denied a history of trauma. On physical examination, there was a limited abduction up to 65 degrees, forward flexion up to 60 degrees, external rotation up to 5 degrees, and internal rotation up to 15 degrees.

Plain radiographs showed again a bulky, bilobed, incongruent osteocartilagenous overgrowth of the proximal humeral epiphysis, but it was asymmetric with exuberant enlargement of the medial component, which presented osteolytic areas and areas of cortical breach (Figure 1B). The cortex was thin at the lateral compartment, but areas of cortical thickening were seen at the medial compartment. Both the medial and the lateral components of the enlarged epiphysis contained flocculent cartilaginous calcifications, but the calcifications of the medial side were less demarcated, resembling cotton-wool, compared to the sharply defined and more solid calcifications of the lateral side. Moreover, rings and arcs of calcifications and osteolytic foci were evident in the proximal metaphysis; the abnormal area of the metaphysis presented a thickening of the cortex with periosteal reaction and a wide zone of transition towards the metaphysis (Figure 1B). These findings were consistent with a malignant cartilaginous tumor of the epiphysis extending to the metaphysis.

**Figure 2:**
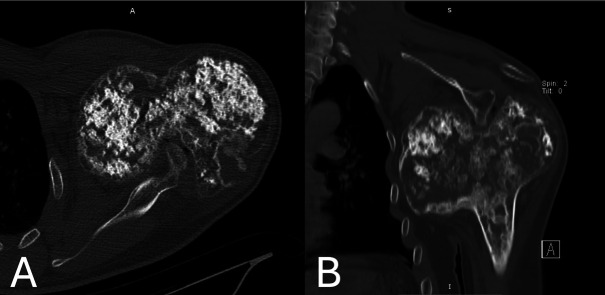
A & B. Computed tomography scan images showing the presence of extensive cartilaginous calcifications, cortical remodeling, and an osteolytic area with cortical disruption at the posterior aspect of the epiphyseal overgrowth. The medial epiphyseal overgrowth extended to the maxillary recess, whereas the glenoid process was seen to indent to the posterior aspect of the epiphysis.

Computed tomography (CT) scan confirmed the presence of extensive cartilaginous calcifications, cortical remodeling, and an osteolytic area with cortical disruption at the posterior aspect of the epiphyseal overgrowth (Figures 2A, 2B).

**Figure 3:**
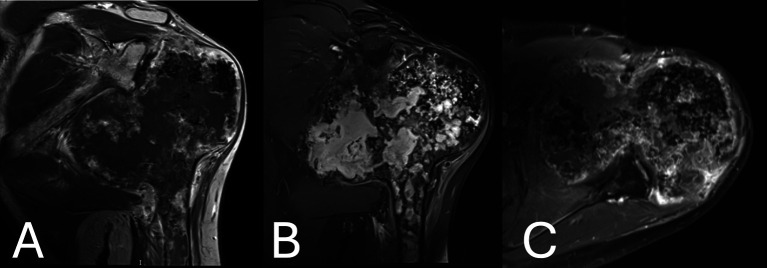
(A) Magnetic resonance imaging showing the exuberant medial compartment, denoted as a large lobulated area, extending to the cortex, with low signal intensity on T1W. (B) Magnetic resonance imaging showing High signal intensity on fat-suppressed T2 W images of the lobulated area on the MRI. Flocculent stippled areas of a signal void, representing cartilaginous calcifications, were more prominent in the lateral compartment. Geographic areas of marrow signal, which are high on T1W and low on fat-suppressed T2W, were entrapped within the cartilaginous tissue of the medial compartment, whereas bone marrow signal was seen at the periphery of the lateral compartment. Cortical disruption was seen at the posterior aspect of the epiphysis corresponding to the osteolytic areas shown on CT. (C) Magnetic resonance imaging post gadolinium administration showing mild peripheral inhomogeneous enhancement. Tissue enhancement was more diffuse or nodular and somewhat more intense at the posterior aspect adjacent to the osteolytic area. Lobules of cartilaginous signal intensity were also seen in the middle and lateral components of the epiphysis, extending to the metaphysis.

On magnetic resonance imaging (MRI), the exuberant medial compartment denoted a large lobulated area, extending to the cortex, with low signal intensity on T1W (Figure 3A) and high signal intensity on fat-suppressed T2W images (Figure 3B) consistent with hyaline cartilage . The osteocartilagenous epiphysis showed mild peripheral inhomogeneous enhancement after gadolinium administration, whereas tissue enhancement was more diffuse or nodular and somewhat more intense at the posterior part aspect adjacent to the osteolytic area, suggesting a higher-grade tumor (Figure 3C). Cortical remodeling, thickening, and mild periosteal reaction of the adjacent metaphysis were also seen, similar to the plain radiographs. The exuberant medial compartment extended below the glenoid process and the lateral border of the scapula, compressing the teres major, the long head of triceps muscles, and the posterior circumflex artery. The glenohumeral joint was incongruent while remodeling of the glenoid process, which indenting to the posterior-superior aspect of the epiphysis was seen.

**Figure 4:**
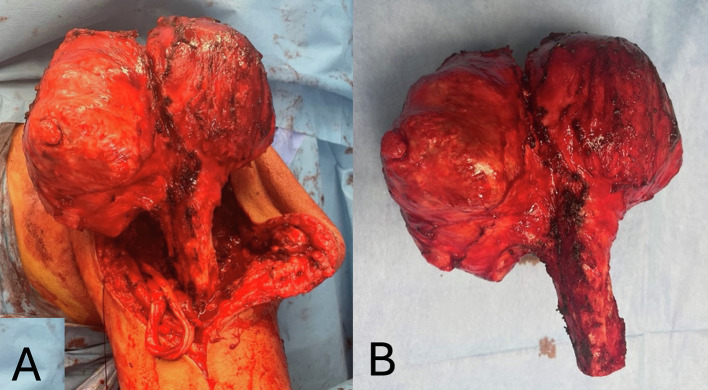
(A) Intra-operative view of the proximal humerus dissected up to the level of the pre-operative agreed safe margin. (B) Specimen resected as planned.

**Figure 5:**
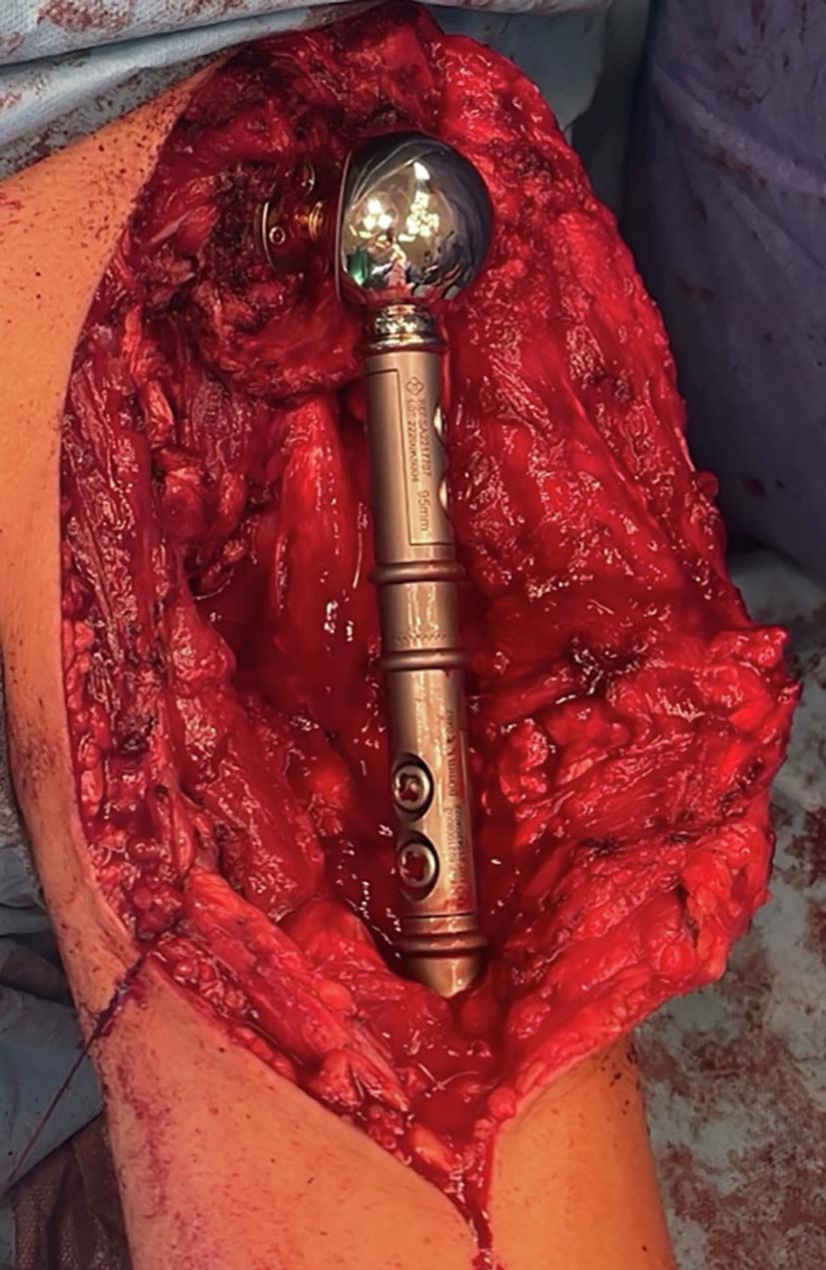
Proximal humerus replacement and constrained reverse total shoulder arthroplasty.

Based on the recent change of the patient’s symptoms, mass size, discomfort and the imaging findings, a wide resection of the proximal humerus and reconstruction has been recommended. The patient underwent a wide resection of the proximal humerus in a beach chair position by the senior author (PJP). The enlarged proximal humerus was apparent and easily palpable. A typical extended deltopectoral approach was undertaken over the palpable mass. Meticulous dissection was performed following the oncological principles, and a distal osteotomy was performed at the level where as per the MRI, no evidence of disease was noted, and a safe margin was agreed to be at that level (Figure 4). The reconstruction was performed with a reverse total shoulder arthroplasty and proximal humeral replacement using a locking glenoid-head mechanism (Implantcast, GmbH, Buxtehude, Germany) (Figure 5).

**Figure 6:**
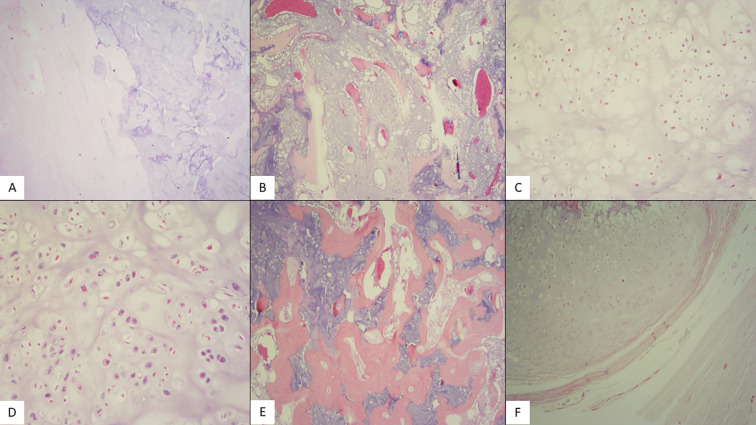
(A) Hematoxylin-eosin stain (X20): Low power shows compact bone infiltration by a chondroid neoplasm. (B) Hematoxylin-eosin stain (X40): Hyaline cartilage lobules expand between spongy bone trabeculae. (C) Hematoxylin-eosin stain (X100): The tumor is composed of hyaline cartilage lobules with mild cellular atypia (chondrosarcoma grade 1) and (D) Hematoxylin-eosin stain (X100): Medium cellular atypia (chondrosarcoma grade 2). Some neoplastic cells are binucleated. Mitoses are rare. (E) hematoxylin-eosin stain (X40): Evident infiltration of the haversian system. (F) Hematoxylin-eosin stain (X40): Extension to periosteal soft tissue.

Macroscopically, the excised lesion was a bulky mass with a maximum diameter of 18 cm, replacing the head and diaphysis of the humerus. Microscopically, the tumor is composed of hyaline cartilage lobules with mild or medium cellular atypia. Some neoplastic cells are binucleated, mitoses are rare, and foci of myxoid degeneration are noted. The tumor infiltrates cancellous and compact bone and focally permeates the latter, eliciting periosteal reaction. Peripheral excision margins are clear, but the tumor infiltrates diaphyseal excision margins (Figures 6A–6F).

**Figure 7:**
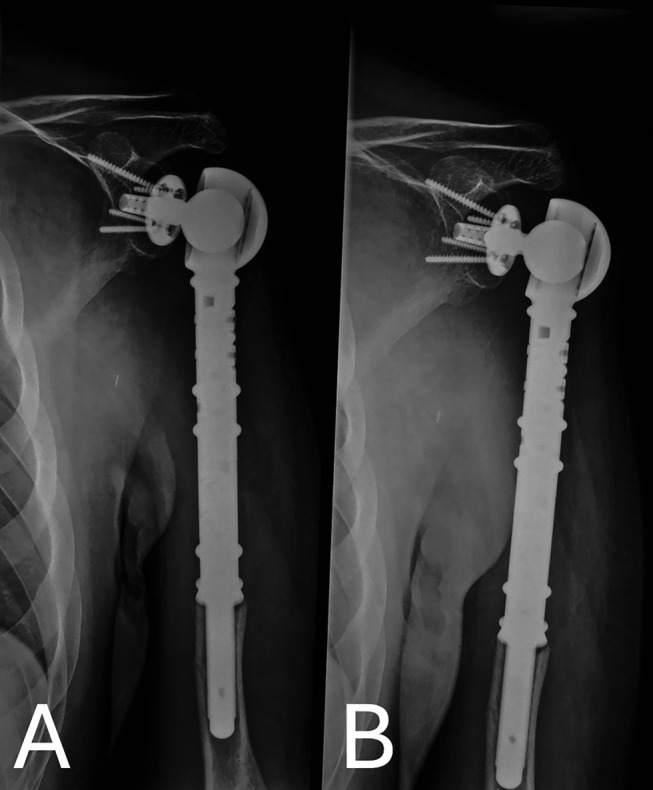
Plain radiograph of the left shoulder at 3.5 years post-operatively showing no signs of loosening or mechanical failure.

The patient has been followed regularly. He is asymptomatic with no signs of local or systemic recurrence at his latest 3.5-year follow-up visit (Figure 7).

## Discussion

Dysplasia Epiphysealis Hemimelica is a rare, sporadic, osteocartilaginous proliferation of not well-understood etiology, primarily affecting the epiphyseal area of long bones in early childhood [2]. Abnormal epiphyseal cartilage growth leads to disorganized ossification, which can result in joint and long bone deformities that may clinically result in a restricted range of motion.

Surgical intervention may be an option depending on the symptomatology and behavior of the disease, either at an early stage to potentially restore normal growth of the physis if possible or to enhance and restore mechanical properties and behavior. Clinical examination, as long as imaging and biopsy are of paramount importance.

We present a case of DEH of the proximal humerus that was followed up for over thirty years until transformation in the clinical and imaging appearance of the enlarged shoulder mandated the need for further intervention. Although the case was diagnosed after biopsy, mentioning the imaging differential diagnosis that includes bulky epiphyseal cartilaginous tumors such as clear cell chondrosarcoma and atypical giant chondroblastoma is rather important. Furthermore, the Epiphyseal giant cell tumor which is also part of the differential diagnosis, does not extend till the periphery of the epiphysis [4], whereas the epiphyseal location of fibrous dysplasia occurs in the polyostotic type of fibrous dysplasia [5]. This is among the very few cases of osteocartilaginous lesions with such a prolonged follow-up period which has transformed to chondrosarcoma.

Such cases must be closely followed up to ensure that the lesions do not manifest any abnormal characteristics, contrary to the low possibility of atypical cartilaginous changes. Regular imaging with plain radiographs or MRI should be obtained to detect signs of malignant transformation, such as increased growth, changes in the morphology of cartilage or bone, abnormal signs of cartilage calcification or ossification alongside clinical signs of aggressive behavior such as emerging pain or swelling in the affected area. Chondrosarcoma can develop as a secondary neoplasm from cartilaginous lesions, including osteochondroma, where the risk of malignant transformation multiplies in the presence of multiple exostoses, or enchondroma, such as in cases of Ollier’s disease or Maffucci’s syndrome.

Malignant transformation in cases of DEH has never been reported before, to the best of our knowledge. A possible explanation for further studies should be identified among factors that alter the cartilage growth patterns, including but not limited to genetic mutations. Prolonged follow-up and patient education for self-monitoring are highly suggested to detect indications of probable transformation.

## Conclusion

This case highlights the need for long-term follow-up in patients with DEH, especially when new symptoms suggest possible malignant transformation.

## Data Availability

The Data are available from the corresponding author upon reasonable request.
